# Supplementary material for: Erratum
Source: Rev Bras Epidemiol. 2024 Sep 30;27:e220002erratum. doi: 10.1590/1980-549720220002.supl.2erratum (PMC11495509; doi:10.1590/1980-549720220002.supl.2erratum)
Supplement: Related texts

# Correction: Errata

**DOI:** 10.1590/1980-549720220002.supl.2.1erratum

https://doi.org/10.1590/1980-549720220002.supl.2.1erratum

No artigo "**Projeto Saúde Brumadinho: aspectos metodológicos e perfil epidemiológico dos participantes da linha de base da coorte**", DOI: https://doi.org/10.1590/1980-549720220002.supl.2.1, publicado no periódico Rev Bras Epidemiol. 2022; 25:e220002.supl.2

**Na página 1 foi incluído:**

EDITOR CIENTÍFICO: Antonio Fernando Boing http://orcid.org/0000-0001-9331-1550
